# Maternal Mortality in Brazil: Proposals and Strategies for its Reduction

**DOI:** 10.1055/s-0038-1672181

**Published:** 2018-09

**Authors:** Rodolfo Carvalho Pacagnella, Marcos Nakamura-Pereira, Flavia Gomes-Sponholz, Regina Amélia Lopes Pessoa de Aguiar, Gláucia Virginia de Queiroz Lins Guerra, Carmen Simone Grilo Diniz, Brenno Belazi Nery de Souza Campos, Eliana Martorano Amaral, Olímpio Barbosa de Moraes Filho

**Affiliations:** 1 Universidade Estadual de Campinas, Campinas, SP, Brazil; 2 Instituto Nacional de Saúde da Mulher, da Criança e do Adolescente Fernandes Figueira, Rio de Janeiro, RJ, Brazil; 3 School of Nursing, Universidade de São Paulo, Ribeirão Preto, SP, Brazil; 4 Universidade Federal de Minas Gerais, Belo Horizonte, MG, Brazil; 5 Instituto de Medicina Integral Professor Fernando Figueira, Recife, PE, Brazil; 6 Universidade de São Paulo, São Paulo, SP, Brazil; 7 Faculdade de Medicina São Leopoldo Mandic, Campinas, SP, Brazil; 8 Universidade de Pernambuco, Recife, PE, Brazil

Maternal mortality is one of the health indicators that most reflect the social conditions of women.^1^ The inequities observed in this indicator between high- and low-income countries and among regions in the same country are explained by differences in the provision, in the access, and in the quality of obstetric care and of family planning. Thus, the death of a woman due to preventable causes is an indicator of her status in society and of the inadequacy of the healthcare system to meet her needs.^2^

The entire health system needs to be ready to respond to the demands of pregnant and postpartum women. Even when adequate prenatal care is performed, it is not possible to predict, as would be ideal, which women will need emergency medical care during labor. Even though it contributes to safer pregnancy and birth, prenatal screening cannot do much to directly impact maternal mortality, because a large proportion of severe complications occur in women who do not present identifiable risk factors during pregnancy.^3^

Thus, even though adequate prenatal care is essential to provide quality care, to manage risk conditions, and to reduce maternal and perinatal morbidity, it is not capable by itself to reduce maternal mortality.^4^ Furthermore, maternal mortality rates are extremely sensitive to adequate obstetric care institutions when complications emerge.^5^ It is not enough to invest in primary prenatal care; systemic actions are also necessary to qualify the emergency care and ensure access to these services. This requires well-trained teams and prompt services to perform the necessary clinical actions whenever diseases or complications manifest.

The interval between the onset of severe obstetric complications and death is estimated at 2 to 6 hours for postpartum hemorrhages and at up to 6 days in case of infections.^1^ ^6^ ^7^ The deaths of pregnant women occur due to delays in obtaining adequate care.

Delays between the onset of a complication and its outcome can take place in one of three phases: I – delayed decision to seek care by individuals and/or their family; II – delayed arrival at a healthcare facility that can provide adequate care; and III – delay in the provision of the necessary care at the necessary time at the institution of reference. All delays are inter-related, and most maternal deaths are caused by a combination of these factors.^8^

In Brazil, maternal death is primarily associated with phases II and III, that is, delay in transportation to higher complexity facilities, and delay in the provision of adequate treatment at healthcare institutions.^9^ Associated with the failure to recognize the signs of severe complications and delayed access to adequate care, a considerable part of maternal deaths that occur even at the tertiary level could be avoided through prompt care in obstetric intensive care units (ICUs) or even in intermediary care units.^10^ ^11^

A significant part of maternal deaths occur after the patient has been admitted in the ICU, and this percentage appears to be on the rise.^11^ Some of these deaths inevitably occur even at institutions that provide the best care available. However, others can be avoided with prompt and structured care delivered by skilled teams.^12^ Implementing the routine use of structured, simple and practical tools for early detection of clinical worsening can help curb maternal mortality. Some examples include the Modified Early Obstetric Warning System (MEOWS) and the structuring of quick responses,^13^ which are strategies that can be applied both in low-complexity hospital environments (such as in maternity wards) and in high-complexity hospital environments (such as in ICUs) by trained multidisciplinary teams.

However, the maternity care network of Brazil presents significant structural gaps that can result in delayed and inadequate care. For example, only 15% of the public maternities have maternal ICUs.^14^ Furthermore, the literature shows that reference facilities should be no farther than 20 km away and that smaller maternities with few patients should be avoided when composing the healthcare system, as they present worse perinatal outcomes.^15^

A population study on births in Brazil^14^ showed that 10% to 24% of low-risk maternities were considered inadequate in terms of physical structure, support services, and human resources to provide quality care. Another example of this inadequacy was presented in a study that observed that obstetricians did not know how to use magnesium sulfate, a drug that is essential to the treatment of hypertensive emergencies in pregnancy, the leading cause of maternal death in Brazil.^16^ ^17^

Two nationwide studies point to flaws in care associated with maternal death in Brazil: the Network for Surveillance of Severe Maternal Morbidity^18^ and the Birth in Brazil study.^19^ These investigations show that maternal deaths or near-misses caused by pregnancy-related complications were associated with barriers to access to specific healthcare services and with inadequate monitoring of complications in the hospital setting.^9^ ^20^ ^21^

However, reducing maternal mortality requires that it should be understood as a form of negligence. It is important to recognize the inequities in the provision of adequate maternity care. In obstetrics, the care relationship is inverted: those who most need care receive it the least, and those who do not require much care receive the most. This creates a paradox, because excessive care and examinations can sometimes be inadequate and even incur unexpected risks.^19^

The greatest challenge is perhaps that of establishing the principle of equity associated with adequate obstetric care. This requires skilled and evidence-based prenatal care. The universal implementation of some measures during prenatal care can prevent several maternal complications and eventual deaths. These actions include:

1) Identifying risk factors at the onset of prenatal care and referring women to specialized services within the appropriate timeframe and ensuring continuity of care.
2) Screening with urine culture tests and antibiograms and providing adequate treatments that do not prolong urinary tract infections.
3) Identifying anemia and providing adequate treatment, ensuring that hemoglobin levels are > 11 g/dL at birth.
4) Preventing high blood pressure by giving acetylsalicylic acid and calcium during the pregnancy for women at risk of preeclampsia.
5) Identifying early signs and symptoms of high blood pressure, such as excessive and sudden weight gain, edema (especially on the face and on the hands), and presence of proteinuria in screening urine tests at the office/outpatient clinic, and early and prompt referral of women with an onset of hypertension in the second half of the pregnancy, especially when associated with proteinuria.
6) Screening for syphilis and HIV at the onset of prenatal care and in the 3^rd^ trimester, and immediate treatment when maternal infection is confirmed.
7) Evaluating and treating vaginal discharge in the earliest phases of pregnancy, before 20 weeks.
8) Previously identifying and planning delivery for higher-risk cases, such as placenta previa, or for women with a history of postpartum hemorrhage.
9) Providing prenatal education regarding low risk vaginal births.
10) Paying special attention to the placental location in women with uterine scars from previous cesarean deliveries and referral to a specialized facility in suspected cases of placenta accreta.

The preventive potential of the measures listed above is easily observed in Brazilian statistics. Pre-eclampsia and eclampsia, postpartum hemorrhage, infections, and unsafe abortion complications represent almost 75% of all maternal deaths. Other causes involve complications from pre-existing diseases (such as heart disease) that are worsened during pregnancy, especially if not managed as part of the prenatal care. In these cases, having access to specialized prenatal care and to an adequate referral system is essential to reduce the risk of death. Additionally, preconception evaluation and reproductive counseling through family planning greatly minimize the risks of pregnancy and delivery.

In Brazil, there is an unmet demand for contraceptives, estimated at 7.7%, which affects between ∼3.5 to 4.2 million women of reproductive age. Of the total number of births in the last five years, only 54% were planned, and unintentional pregnancies were reported by 55.4% of postpartum women.^22^ ^23^ ^24^ ^25^ Unwanted pregnancies increase the risk of maternal mortality.

Women who are more socially vulnerable (which is characterized by young maternal age, unemployment, alcohol abuse, and higher number of children) present higher rates of unwanted pregnancies. Women with more than three children are 14 times more likely to have an unwanted pregnancy. Furthermore, complications in previous pregnancies or premature births also increase by 40% the chances of an unplanned pregnancy.^25^ These numbers are a reflection of the lack of access to family planning and skilled care before, during, and after delivery. Because of these high numbers of unwanted pregnancies, these women frequently resort to unsafe abortion practices.

The unsafe termination of pregnancy (which is usually performed by untrained individuals, with dangerous instruments or in unhygienic institutions) is another relevant aspect regarding family planning that strongly impacts public health.^26^ Unsafe abortions are the main direct cause of maternal death in Brazil. The complications caused by this type of procedure represent the third leading cause of obstetric bed occupancy in Brazil.

Countries with more restrictive laws for terminating pregnancy present higher rates of maternal mortality due to abortion-related causes, result of a higher number of unwanted pregnancies. A perverse aspect of abortions is that lower-income women are more vulnerable to unsafe and clandestine procedures, while women with a higher economic standing have access to safer terminations, albeit still clandestine, with fewer chances of complications and death.

For these reasons, from the perspective of improving the quality of healthcare, medical organizations agree with the proposal still being analyzed by the Brazilian National Congress to legalize the termination of pregnancy in the following situations (**NATIONAL COUNCIL OF MEDICINE NEWSLETTER No. 46/2013)**:

- When the “woman's life or health is at risk”;
- When “the pregnancy is a result of rape or of the nonconsensual use of assisted reproduction techniques”;
- When “anencephaly is proven or when the fetus suffers from severe and incurable abnormalities that prevent independent life, in both cases with a report signed by two physicians”; and
- When “the woman desires to terminate before the 12^th^ week of pregnancy”.

In 2009, the Human Rights Council of the United Nations recognized maternal mortality as an issue bearing on human rights, and that its prevention is immersed in a challenging political context that, at times, prevents or hinders change.^27^ ^28^ When maternal mortality is viewed as a form of negligence, it is easier to hold governments accountable for health, education, and development policies, as well as for international agreements of which they are signatories to ensure basic human rights, which include good health conditions for women and a safe motherhood.^29^

Despite numerous advances in reducing the incidence of maternal and child death in the past 30 years, with the creation of the Brazilian Unified Health System (SUS, in the Portuguese acronym),^30^ Brazil has not yet reached the Millennium Development Goal 5 of reducing its maternal mortality rate to 35 deaths per 100,000 live births by 2015. The country is supposed to achieve an even greater goal by 2030, on the renegotiation of the Sustainable Development Goals, an international commitment taken on by the country. According to this document, Brazil should reduce the maternal mortality rate, currently arround 60 deaths per 100,000 live births, to 20 per 100,000 between 2015 and 2030.

Between 1990 and 2015, the maternal mortality rate decreased 56%, falling from 143 to 62 deaths per 100,000 live births. However, since 2000, this downward trend has been slowing down, despite the increase in maternal care coverage.^31^ The Ministry of Health recognizes the great challenge it must overcome and has recently considered whether it is in conditions to reach the maternal mortality rate of 30 per 100,000 live births by 2030. For this reason, on May 31, 2018, the Brazilian Ministry of Health instituted the I National Mobilization Week for Women's Health in the SUS, which had as its opening theme the reduction of maternal mortality.

The Ministry of Health proposed strengthening actions already in progress and listed several developed actions that can be reviewed to address this objective, such as the Rede Cegonha (Stork Network), birth centers, midwifes in obstetric care, expanding the supply of contraceptives, and especially the implementation of programs for postpartum and postabortion insertion of intrauterine devices (IUDs), as well as specific care for vulnerable populations, and projects to improve the quality of obstetric care.

However, to reach this goal, differences among states must be considered, in addition to differences in maternal mortality between populations of different ethnicities. It is also essential to advance in the recognition of the problem of maternal near-misses and the need for strategies to reduce maternal mortality. Furthermore, it is necessary to strengthen the line of care provided to women in the pregnancy-puerperal cycle by identifying technical and structural flaws in the obstetric care provided in Brazil.

A considerable part of the problem of maternal mortality occurs because of the disorganization of the care networks, especially in terms of the lack of clear care protocols to manage severe conditions that result in maternal death. There are four triggering causes of maternal death that account for practically all maternal mortality in the country: preeclampsia, postpartum hemorrhage, sepsis, and unsafe abortions. Thus, it is essential to qualify services to provide care in these conditions.

However, reducing maternal mortality requires systemic action, in which all levels of care and all points of the health line of care for women are considered, from contraception to the postpartum follow-up. Brazil presents several hypermedication paradoxes (reflected in unprecedented cesarean birth rates) and insufficient skilled human resources. There is a wide array of practices, risk factors, and determinants that result in the death of women in Brazil: lack of adequate human resources, medications, emergency care equipment in many regions, and lack of evidence-based care during labor.^23^ When using evidence-based practices, reducing interventions during labor has been shown to reduce maternal morbidity: for example, intrapartum oxytocin has been associated with severe postpartum hemorrhaging,^32^ and cesarean delivery is considered a risk factor for maternal death.^33^

The urgent issue of reducing the delay in the access to adequate obstetric care in Brazil can be addressed through simple measures. Thus, considering that the main causes of maternal mortality are preventable, and that their determinants and constraints are directly related to the lack of adequate care, actions to reduce maternal mortality in Brazil must be based on the assumptions that:

1. Maternal and perinatal health is an issue for national development.
2. Local and state investigation committees of maternal mortality and near-misses must be supported.
3. Women need access to skilled and comprehensive care from the technical and humanistic point of view before, during, and after the pregnancy and labor, including:
  - Access to reproductive planning that meets their personal needs, respecting cultural and religious differences, promoting effective actions to increase the use of long-acting methods (IUDs and implants).
  - Access to quality preconception planning that promotes safe and planned pregnancies.
  - Effective, safe, and reversible reproductive planning, including long-acting methods (IUDs and implants) and actions to provide universal access to postpartum and postabortion IUD insertion.
  - Early detection of pregnancy, with the systematic adoption of the practices covered in the technical guidelines provided by the Ministry of Health.
  - Early and skilled prenatal care to identify and manage social and clinical conditions that represent a risk for complications during pregnancy, labor and postpartum.
  - Connection between prenatal care services and delivery facilities.
  - Houses for pregnant women (*Casas da Gestante*) with easy access to hospital services for women who present with complications or are at a higher risk of complications and do not have easy access to transportation or who live in remote areas.
  - Quality multidisciplinary care during labor that abides by the women's wishes and expectations and is in line with safety protocols for the mother, the fetus, and the newborn.
  - Access to safe abortions, respecting the women's wishes and embracing their needs, in cases provided for by the law.
4. Obstetric care networks include:
  - Institutions with adequate structure to perform cesarean deliveries, emergency surgical procedures, and access to prompt hemotherapy (blood banks and/or transfusion services) at all its points of care.
  - A regional organization in which small maternities have limited importance, and mid-sized hospitals that can meet this demand receive more resources.
  - Skilled and experienced teams for both low and high complexity situations.
  - Access to the main drugs needed to immediately treat complications that lead to maternal death at all levels of the obstetric network, including emergency care services (magnesium sulfate, nifedipine, venous hydralazine, oxytocin, misoprostol, and antibiotics).
  - The following services:
    - Prenatal care for low- and high-risk pregnancies, which are interconnected and connected with a hospital care network.
    - Minimum screening laboratory tests or others depending on the condition of the pregnant woman, according to the national protocols.
    - Birth centers and low-risk maternities with quick access to emergency care, including anesthesia and timely access to laboratory tests and hemotherapy (blood banks and/or transfusion services).
    - Maternities of reference for high-risk cases.
    - Obstetric ICUs in high-risk maternities.
    - An efficient system for referrals and for regulating occupancy rates.
    - Adequate and agile transportation system for referrals.
    - Obstetric ICU beds, and when maternities do not have ICUs, they have agreements with general hospital ICUs to prevent any delay in transfers.
  - Skilled and legally certified obstetric care teams according to level of care, which include:
    - Obstetric nurses/midwifes
    - Obstetricians
    - Anesthesiologists
    - Intensive care physicians
5. Health professionals must be trained in routine and emergency obstetric care, with the support of class entities, professional associations, universities, and health managers, focusing on:
  - In-person and e-learning refresher courses about evidence-based maternal care
  - Up-to-date care protocols for more severe obstetric situations
    - Preeclampsia and eclampsia
    - Hemorrhages, with transfusion protocols and adequate postpartum hemorrhage management.
    - Maternal sepsis
    - Vaginal and cesarean deliveries
    - Safe abortions
  - Continuing education programs for maternities to adequately manage vaginal and cesarean deliveries and to identify and manage severe obstetric conditions.
  - Medical residency, multidisciplinary residency, specialization courses, and undergraduate training centered on the basic principles of prenatal, birth, and postpartum care, including care during normal physiological birth and strategies to reduce interventions in the absence of clinical indications (inductions, oxytocin, cesareans, etc.).
  - Auditing programs in cases of severe maternal and perinatal morbidity in maternities and in health regions, in addition to the frequency of interventions and related outcomes (cesareans, episiotomies, use of medications for induction/conduction/augmentation, etc.).
6. Social mobilization as a form of reducing maternal and perinatal mortality, including:
  - Professional and well-informed reproductive rights advocacy groups.
  - Ongoing campaigns by the media, schools, communities, and health professional education about the quality of women's health care as an indicator of inequities and sustainable development.
  - Education and capacity-building programs for adult and adolescent women about women's health, including pregnancy, birth and postpartum.
  - Investment in formal and informal education of girls and young women to provide better life options.
  - Guaranteed regular investment in maternal health by the competent ministries.
  - The integration of intersectoral actions, with emphasis on women's health, based on the Sustainable Development Goals.
  - Debates involving lawmakers and legal experts with greater involvement of the Public Prosecutor's Office to inspect the adequate functioning of health services and to discuss the expansion of the right to abortion as per the National Medical Council (CFM, in the Portuguese acronym) Newsletter no. 46/2013, and to support the act of noncompliance of a fundamental obligation (ADPF, in the Portuguese Acronym) petition 442 that is currently moving through the Federal Supreme Court.

Brazil has a history of innovative public policies for women. However, reducing maternal morbimortality indicators still requires specific government attention. The implementation of these policies must be reformulated. Even though the theoretical conception of these policies is comprehensive, the health care provided to pregnant women is still precarious in several contexts in Brazil. Today, the role of obstetric care services in reducing maternal mortality is undeniable. Maternal death cannot be attributed to one single factor; therefore, actions must be formulated systematically if they are to enable the reduction of such high maternal mortality rates.

## Notes

**Erratum:** The affilations of DOI: 10.1055/s-0038-1672181 has been changed from Brenno Belazi Nery de Souza Campos^7^ Eliana Martorano Amaral^1,7^ to Brenno Belazi Nery de Souza Campos^1,7^ Eliana Martorano Amaral^1^.

## References

[1] Maine D *Safe Motherhood Programs: Options and Issues* New York, NY Columbia University 1993

[2] World Health Organization, Maternal Health and Safe Motherhood Programme. *Mother-Baby Package: Implementing Safe Motherhood in Countries* Geneva World Health Organization 1996

[3] Rosenfield A Maine D Maternal mortality--a neglected tragedy. Where is the M in MCH? Lancet 1985 2(8446):83 85. Doi: 10.1016/S0140-6736(85)90188-6 2861534 10.1016/s0140-6736(85)90188-6

[4] Paxton A Maine D Freedman L Fry D Lobis S The evidence for emergency obstetric care Int J Gynaecol Obstet 2005 88 02 181 193. Doi: 10.1016/j.ijgo.2004.11.026 15694106 10.1016/j.ijgo.2004.11.026

[5] Loudon I Obstetric care, social class, and maternal mortality Br Med J (Clin Res Ed) 1986 293(6547):606 608. Doi: 10.1136/bmj.293.6547.606 10.1136/bmj.293.6547.606 PMC1341393 3092949

[6] Ganatra B R Coyaji K J Rao V N Too far, too little, too late: a community-based case-control study of maternal mortality in rural west Maharashtra, India Bull World Health Organ 1998 76 06 591 598 http://www.pubmedcentral.nih.gov/articlerender.fcgi?artid=2312494&tool=pmcentrez&rendertype=abstract. Accessed March 20, 2017 10191555 PMC2312494

[7] Grimes D A Benson J Singh S Unsafe abortion: the preventable pandemic Lancet 2006 368(9550):1908 1919. Doi: 10.1016/S0140-6736(06)69481-6 17126724 10.1016/S0140-6736(06)69481-6

[8] Thaddeus S Maine D Too far to walk: maternal mortality in context Soc Sci Med 1994 38 08 1091 1110. Doi: 10.1016/0277-9536(94)90226-7 8042057 10.1016/0277-9536(94)90226-7

[9] Pacagnella R C Cecatti J G Parpinelli M A Delays in receiving obstetric care and poor maternal outcomes: results from a national multicentre cross-sectional study BMC Pregnancy Childbirth 2014 14 159. Doi: 10.1186/1471-2393-14-159 24886330 10.1186/1471-2393-14-159 PMC4016777

[10] Pollock W Rose L Dennis C L Pregnant and postpartum admissions to the intensive care unit: a systematic review Intensive Care Med 2010 36 09 1465 1474. Doi: 10.1007/s00134-010-1951-0 20631987 10.1007/s00134-010-1951-0

[11] Oud L Epidemiology of pregnancy-associated ICU utilization in Texas: 2001–2010 J Clin Med Res 2017 9 02 143 153. Doi: 10.14740/jocmr2854w 28090230 10.14740/jocmr2854w PMC5215018

[12] Cantwell R Clutton-Brock T Cooper G Saving mothers' lives: reviewing maternal deaths to make motherhood safer: 2006–2008. The Eighth Report of the Confidential Enquiries into Maternal Deaths in the United Kingdom BJOG 2011 118 01 1 203. Doi: 10.1111/j.1471-0528.2010.02847.x 10.1111/j.1471-0528.2010.02847.x 21356004

[13] Shamshirsaz A A Dildy G A Reducing Maternal Mortality and Severe Maternal Morbidity: The Role of Critical Care Clin Obstet Gynecol 2018 Jun;61 02 359 371. Doi: 0.1097/GRF.0000000000000370 29629925 10.1097/GRF.0000000000000370

[14] Bittencourt S DA Domingues R MSM Reis L GC Ramos M M Leal M D Adequacy of public maternal care services in Brazil Reprod Health 2016 13 03 120. Doi: 10.1186/s12978-016-0229-6 27766964 10.1186/s12978-016-0229-6 PMC5073989

[15] Ministério das Relações Exteriores. *Objetivos de Desenvolvimento Sustentável*. 2017 http://www.itamaraty.gov.br/images/ed_desenvsust/ODSportugues12fev2016.pdf. Accessed July 21, 2018

[16] Lotufo F A Parpinelli M A Osis M J Surita F G Costa M L Cecatti J G Situational analysis of facilitators and barriers to availability and utilization of magnesium sulfate for eclampsia and severe preeclampsia in the public health system in Brazil BMC Pregnancy Childbirth 2016 16 254. Doi: 10.1186/s12884-016-1055-0 27577571 10.1186/s12884-016-1055-0 PMC5006565

[17] Lotufo F A Parpinelli M A Osis M J Surita F G Costa M L Cecatti J G Obstetrician's risk perception on the prescription of magnesium sulfate in severe preeclampsia and eclampsia: A qualitative study in Brazil PLoS One 2017 12 03 e0172602. Doi: 10.1371/journal.pone.0172602 28301493 10.1371/journal.pone.0172602 PMC5354257

[18] Cecatti J G Costa M L Haddad S M Network for Surveillance of Severe Maternal Morbidity: a powerful national collaboration generating data on maternal health outcomes and care BJOG 2016 123 06 946 953. Doi: 10.1111/1471-0528.13614 26412586 10.1111/1471-0528.13614

[19] do Carmo Leal M da Silva A A Dias M AB Birth in Brazil: national survey into labour and birth Reprod Health 2012 9 15. Doi: 10.1186/1742-4755-9-15 22913663 10.1186/1742-4755-9-15 PMC3500713

[20] Domingues R MSM Dias M AB Schilithz A OC Leal M D Factors associated with maternal near miss in childbirth and the postpartum period: findings from the birth in Brazil National Survey, 2011-2012 Reprod Health 2016 13 03 115. Doi: 10.1186/s12978-016-0232-y 27766973 10.1186/s12978-016-0232-y PMC5073804

[21] Giordano J C Parpinelli M A Cecatti J G The burden of eclampsia: results from a multicenter study on surveillance of severe maternal morbidity in Brazil PLoS One 2014 9 05 e97401. Doi: 10.1371/journal.pone.0097401 24825164 10.1371/journal.pone.0097401 PMC4019598

[22] Hart J T The inverse care law Lancet 1971 1(7696):405 412. Doi: 10.1016/S0140-6736(71)92410-X 4100731 10.1016/s0140-6736(71)92410-x

[23] Torloni M R Betrán A P Belizán J M Born in Brazil: shining a light for change Reprod Health 2016 13 01 133. Doi: 10.1186/s12978-016-0247-4 27756402 10.1186/s12978-016-0247-4 PMC5070232

[24] Diniz S G Chacham A S “The cut above” and “the cut below”: the abuse of caesareans and episiotomy in São Paulo, Brazil Reprod Health Matters 2004 12 23 100 110. Doi: 10.1016/S0968-8080(04)23112-3 15242215 10.1016/s0968-8080(04)23112-3

[25] Theme-Filha M M Baldisserotto M L Fraga A CSA Ayers S da Gama S G Leal M D Factors associated with unintended pregnancy in Brazil: cross-sectional results from the Birth in Brazil National Survey, 2011/2012 Reprod Health 2016 13 03 118. Doi: 10.1186/s12978-016-0227-8 27766945 10.1186/s12978-016-0227-8 PMC5073899

[26] Ventura M A mortalidade materna: a persistente violação do direito de proteção da vida e autonomia feminina Rev Bioet 2008 16 217 228

[27] Maine D Detours and shortcuts on the road to maternal mortality reduction Lancet 2007 370(9595):1380 1382. Doi: 10.1016/S0140-6736(07)61580-3 17933653 10.1016/S0140-6736(07)61580-3

[28] Rosenfield A Maine D Freedman L Meeting MDG-5: an impossible dream? Lancet 2006 368(9542):1133 1135. Doi: 10.1016/S0140-6736(06)69386-0 17011925 10.1016/S0140-6736(06)69386-0

[29] AbouZahr C Global burden of maternal death and disability Br Med Bull 2003 67 1 11. Doi: 10.1093/bmb/ldg015 14711750 10.1093/bmb/ldg015

[30] Leal M DC Szwarcwald C L Almeida P VB Saúde reprodutiva, materna, neonatal e infantil nos 30 anos do Sistema Único de Saúde (SUS) Cien Saude Colet 2018 23 06 1915 1928. Doi: 10.1590/1413-81232018236.03942018 29972499 10.1590/1413-81232018236.03942018

[31] Diniz S G d'Oliveira A FPL Lansky S Equity and women's health services for contraception, abortion and childbirth in Brazil Reprod Health Matters 2012 20 40 94 101. Doi: 10.1016/S0968-8080(12)40657-7 23245414 10.1016/S0968-8080(12)40657-7

[32] Belghiti J Kayem G Dupont C Rudigoz R C Bouvier-Colle M H Deneux-Tharaux C Oxytocin during labour and risk of severe postpartum haemorrhage: a population-based, cohort-nested case-control study BMJ Open 2011 1 02 e000514. Doi: 10.1136/bmjopen-2011-000514 10.1136/bmjopen-2011-000514 PMC3334825 22189353

[33] Esteves-Pereira A P Deneux-Tharaux C Nakamura-Pereira M Saucedo M Bouvier-Colle M H Leal MdoC Caesarean delivery and postpartum maternal mortality: a population-based case control study in Brazil PLoS One 2016 11 04 e0153396. Doi: 10.1371/journal.pone.0153396 27073870 10.1371/journal.pone.0153396 PMC4830588

