# Erratum – Maternal Mortality in Brazil: Proposals and Strategies for its Reduction

**DOI:** 10.1055/s-0038-1676846

**Published:** 2019-01

**Authors:** Rodolfo Carvalho Pacagnella, Marcos Nakamura-Pereira, Flavia Gomes-Sponholz, Regina Amélia Lopes Pessoa de Aguiar, Gláucia Virginia de Queiroz Lins Guerra, Carmen Simone Grilo Diniz, Brenno Belazi Nery de Souza Campos, Eliana Martorano Amaral, Olímpio Barbosa de Moraes Filho

**Affiliations:** 1 Universidade Estadual de Campinas, Campinas, SP, Brazil; 2 Instituto Nacional de Saúde da Mulher, da Criança e do Adolescente Fernandes Figueira, Rio de Janeiro, RJ, Brazil; 3 School of Nursing, Universidade de São Paulo, Ribeirão Preto, SP, Brazil; 4 Universidade Federal de Minas Gerais, Belo Horizonte, MG, Brazil; 5 Instituto de Medicina Integral Professor Fernando Figueira, Recife, PE, Brazil; 6 Universidade de São Paulo, São Paulo, SP, Brazil; 7 Faculdade de Medicina São Leopoldo Mandic, Campinas, SP, Brazil; 8 Universidade de Pernambuco, Recife, PE, Brazil

Rio de Janeiro, December 11, 2018

Dear readers,

In the E-Poster *Maternal Mortality in Brazil: Proposals and Strategies for its Reduction* (*Rev Bras Ginecol Obstet 2018;40.09: 501-506_4010ed. DOI:* 10.1055/s-0038-1672181.), published online in *Rev Bras Ginecol Obstet* in September 2018, where it reads:

Brenno Belazi Nery de Souza Campos^7^ Eliana Martorano Amaral^1,7^

It should read:

Brenno Belazi Nery de Souza Campos^1,7^ Eliana Martorano Amaral^1^

